# Seasonal weather and climate prediction over area burned in grasslands of northeast China

**DOI:** 10.1038/s41598-020-76191-2

**Published:** 2020-11-17

**Authors:** Ali Hassan Shabbir, Jiquan Zhang, John W. Groninger, Eddie J. B. van Etten, Samuel Asumadu Sarkodie, James A. Lutz, Carlos Valencia

**Affiliations:** 1 Institute of Natural Disaster Research, School of Environment, Northeast Normal University, Changchun, 130024 China; 2 State Environmental Protection Key Laboratory of Wetland Ecology and Vegetation Restoration, Northeast Normal University, Changchun, 130024 China; 3 Key Laboratory for Vegetation Ecology, Ministry of Education, Changchun, 130024 China; 4 Department of Forestry, Southern Illinois University, Mail Code 4411, Carbondale, IL 62901 USA; 5 Centre for Ecosystem Management, Edith Cowan University, Joondalup, Perth, 6027 Australia; 6 Nord University Business School (HHN), Post Box 1490, 8049 Bodø, Norway; 7 Wildland Resources Department, Utah State University, 5230 Old Main Hill, Logan, UT 84322-5230 USA; 8 Industrial Engineering, University of Los Andes, Cra1 Este 19-40, Bogota, Colombia

**Keywords:** Fire ecology, Climate-change ecology, Grassland ecology

## Abstract

Grassland fire dynamics are subject to myriad climatic, biological, and anthropogenic drivers, thresholds, and feedbacks and therefore do not conform to assumptions of statistical stationarity. The presence of non-stationarity in time series data leads to ambiguous results that can misinform regional-level fire management strategies. This study employs non-stationarity in time series data among multiple variables and multiple intensities using dynamic simulations of autoregressive distributed lag models to elucidate key drivers of climate and ecological change on burned grasslands in Xilingol, China. We used unit root methods to select appropriate estimation methods for further analysis. Using the model estimations, we developed scenarios emulating the effects of instantaneous changes (i.e., shocks) of some significant variables on climate and ecological change. Changes in mean monthly wind speed and maximum temperature produce complex responses on area burned, directly, and through feedback relationships. Our framework addresses interactions among multiple drivers to explain fire and ecosystem responses in grasslands, and how these may be understood and prioritized in different empirical contexts needed to formulate effective fire management policies.

## Introduction

Human-populated grassland ecosystems are especially responsive to interrelated and dynamic physical, social, and biological forces. Climate, human-driven ignition and grazing dynamics, vegetation, and fuel characteristics, all interact to shape fire activity in grassland ecosystems^1–9^. Incidence and seasonality of grassland fire are both associated with natural climate variations and anthropogenic ignition patterns^3,9–12^. The potentially complex interactions among these drivers, along with anthropogenic climate change and the importance of grasslands ecosystems to sustaining regional livelihoods, add urgency to better understanding these relationships over multi-year periods.

Burned grassland extent is influenced by regional climate change through increasing temperature which reduces fuel moisture and, thereby, increases flammability^13–16^, but climate change also alters productivity, biomass abundance, and carbon emissions^17,18^, all factors which drive fuel accumulation dynamics^13,19,20^. Further, effects of changing climates on fire activity may only manifest once critical thresholds are crossed^21,22^. Such feedbacks and thresholds can mediate subsequent ecosystem responses to ecosystem drivers in the face of significant change in the annual extent of burned grassland incidence. Ongoing and coherent short-term change in climate across the Xilingol region provide a responsive example of potential direct anthropogenic and climatic drivers shifting critical ecosystem responses through measurable ecological traits via threshold or non-linear relationships^22^.

To ensure the provision of critical ecosystem services and the viability of grassland- dependent enterprises, ecologists and policymakers need reliable models to better understand the short- and long-term impacts of dynamic climatic and anthropogenic ecosystem drivers^22,23^. Because ecological responses can be relatively insensitive to climate, at least until thresholds are crossed^22^, ecological modeling using ordinary least square regression (OLS) has proven to be of little value to wildfire policymakers and managers^24^. Applicability of these models in grassland ecosystems have been limited by poor connectivity between climatic processes and ecological response for two reasons: First, is the need to understand the error structure which, if not properly estimated, especially in the case of highly skewed and heterogeneous data, can bias parameter estimations that produce false precision^25^. Second, fire management requires appropriate information regarding human interactions with burned grasslands as these may influence seasonality and extent of the fire, and their impacts over time. The issue of stationarity in the dominant fire-climate relationships in time-series data in response to human-caused fire activity has not been well represented in the previous studies^1,4,5,13,23^. Further, this information is often overlooked in summarized fire-climate relationships.

To explore the ability of dynamic simulations to understand and predict fire activity in grasslands, we need to target existing knowledge gaps by comparing trends in climatically driven changes in grassland fire activity and confirm that the outcomes are indeed consistent with our expectations of the key disturbance drivers of climate and ecological change on burned grasslands. To do this, we must first apply unit root tests to resolve non-stationarity issues in time series fire data from anthropogenic grasslands in order to select whether Johansen Cointegration^26^, OLS, or dynamic simulations such as Autoregressive Distributed Lags (ARDL)^27^ models are most appropriate. The reason for selecting the dynamic simulated ARDL model over the others is because it not only addresses complicated interactions among fire-relevant climate variables at different levels but also accounts for dynamic processes and feedbacks in a system. This research also attempts to extract additional measures from the models that can improve system predictability and reliably inform stakeholders. In this study, we aim to clarify the key drivers of climate and ecological change on burned grasslands in Xilingol, China using the novel approach of dynamic simulations of autoregressive distributed lag models.

### Study area and data description

**Figure 1:**
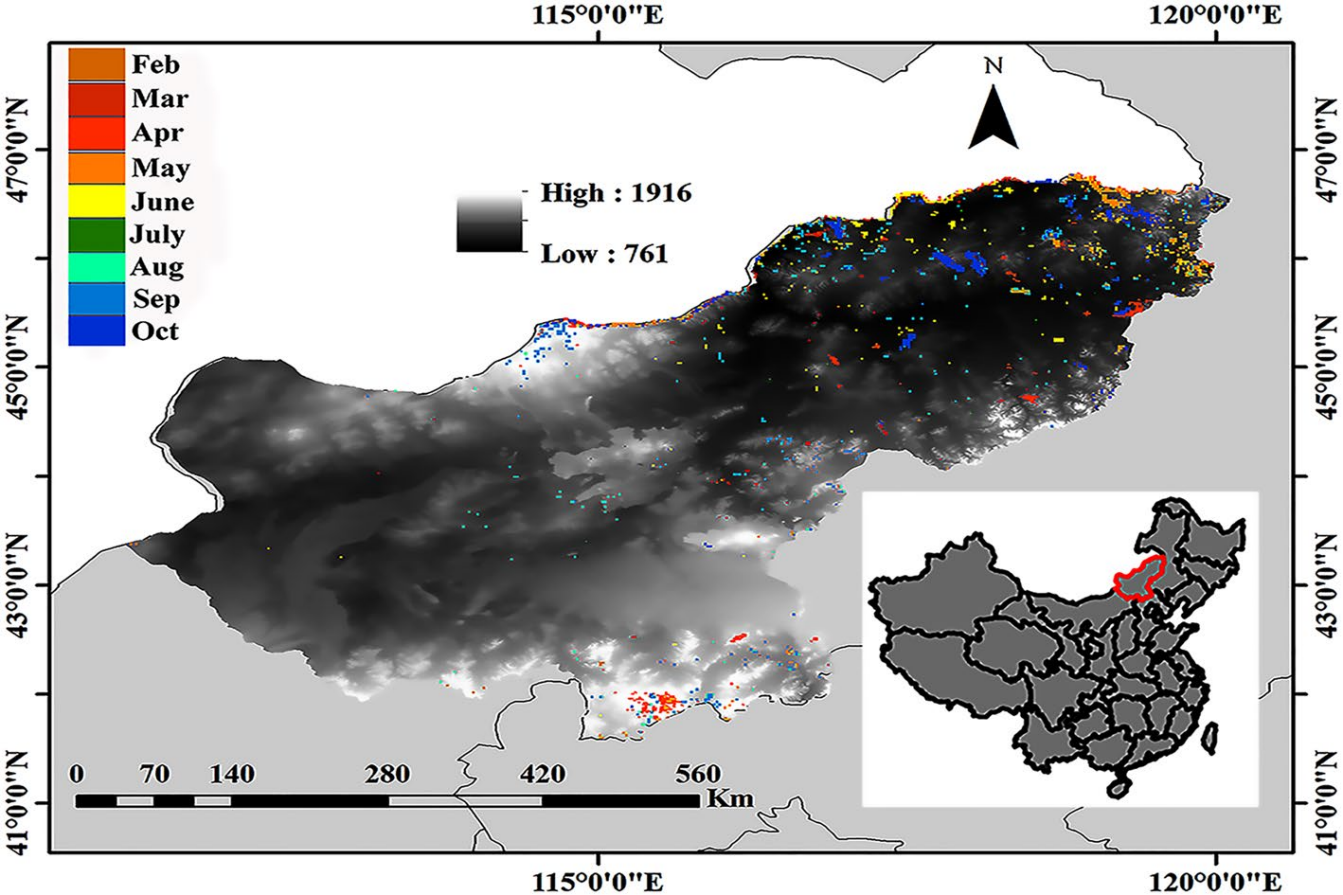
The location of the study area showing burned grasslands in the period 2000–2018 with each color representing a different month. The grey shading represents elevation from 761 to 1916 MAMSL. Map generated with ArcGIS 10.3, (https://desktop.arcgis.com/), with data sourced from {https://lpdaac.usgs.gov/data_access/data_pool}.

**Figure 2:**
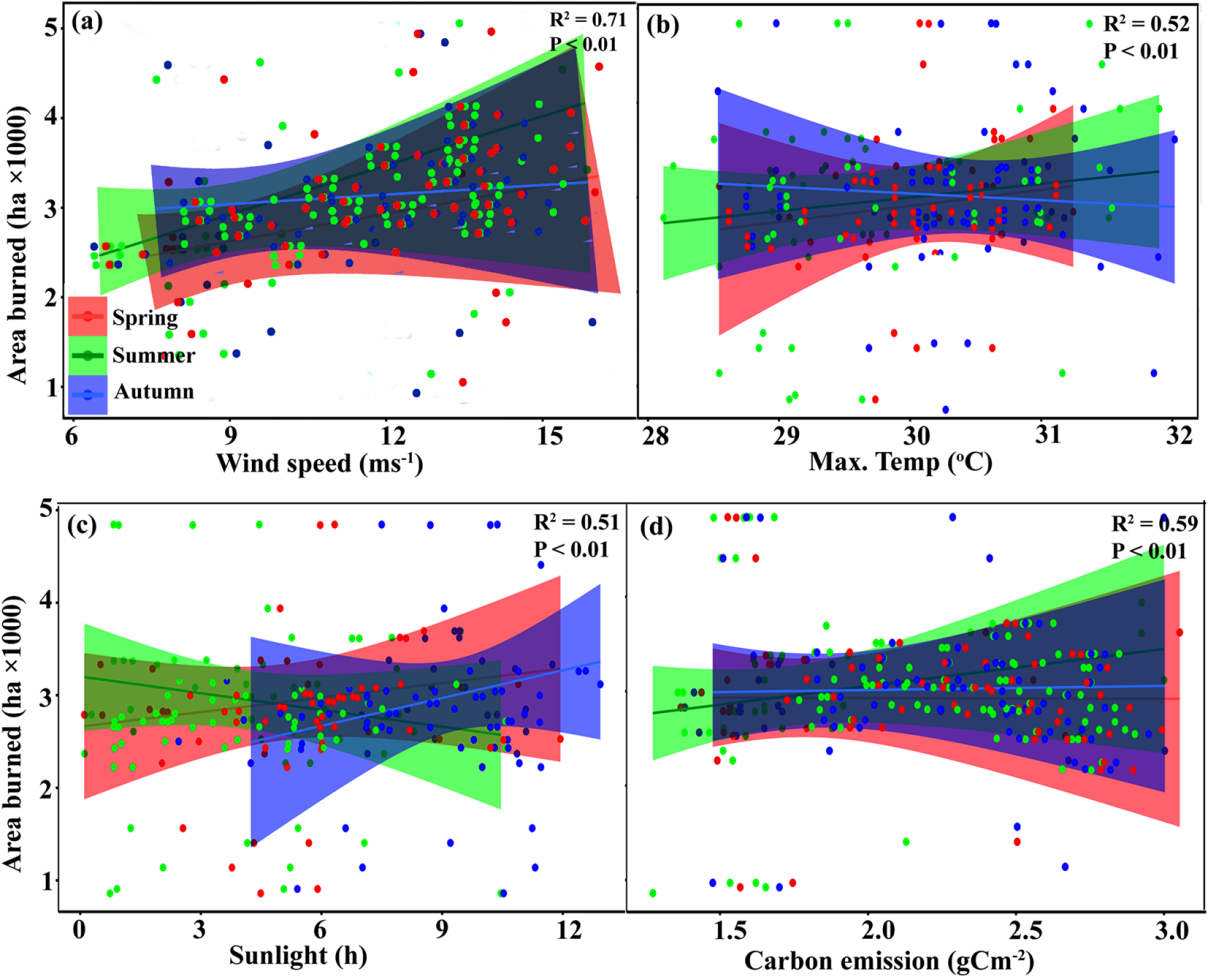
Influence of climate variables on burned grassland in Xilingol. Spring, Summer, and Autumn-burned grasslands are represented in red, green, and blue, respectively.

The Xilingol League lies about 600 km north of Beijing in central Inner Mongolia, the most representative steppe grassland in northern China (Fig. 1). Grassland covers 70% of the total area of 2.03 × 10^7^ ha. Grazing has been the extensive land use in the Xilingol League where climate variations and other factors, including fire-generating human-activities, are the principal contributing factors to increased incidence of grassland burning^28^. Heterogeneous climate and human activities contribute to a complex burn matrix throughout Xilingol. Fire occurrence and severity is greatest from spring through autumn, promoted by drying effects of wind and other climatic drivers (Fig. 2).

We used eight climatic variables to evaluate the effects of key drivers on burned grasslands and ecological change: monthly average maximum temperature (in degrees C); monthly average sunlight (in hours per month); monthly average wind speed (in meters per second); the monthly average carbon emission (in grams per square centimeter^2^); monthly average vapor pressure deficit; monthly average precipitation (in millimeters); and monthly average relative humidity (percentage). Heteroscedasticity^29^, autocorrelation^30^, and multicollinearity^31^ are evident in their effect on the climate records of burned grasslands. We then reanalyzed, assessed the fire-relevant climate variables, and confirmed the trends that are consistent with our expectations.

## Results and discussion

Consistent with best practices for homoscedastic stationary series using small data sets, we used the ADF, PP, KPSS, and DF-GLS tests to confirm model validation (Table 1). Maximum temperature and wind speed were stationary I(0) at a 1% level of significance while other variables were integrated to order one I(1) indicating stationarity at first difference. According to the DF-GLS test, the mean minimum monthly temperature was also stationary I(0). The absence of integration to order two I(2) for any of the variables indicated the suitability of the ARDL approach.

**Table 1:** Unit root test results of climatic variables for Augmented Dickey–Fuller (ADF), Phillips–Perron (PP), Kwiatkowski, Phillips, Schmidt, Shin (KPSS), and modified Dickey-Fuller generalized least squares (DF-GLS).

Variables	ADF		PP		KPSS		DF-GLS	
	Level	1st Diff	Level	1st Diff	Level	1st Diff	Level	1st Diff
ln T_min_	− 7.05	− 19.05**	− 9.91	− 16.08**	0.255	0.052**	− 1.41*	− 16.08**
ln Rel.humidity	− 8.63	− 13.32**	− 8.75	− 24.93**	0.183	0.092**	− 3.05	− 23.63**
ln T_max_	− 9.14*	− 14.41**	− 8.55*	− 12.43**	0.234*	0.011**	− 4.55*	− 23.63**
ln Precip	− 8.96	− 13.21**	− 9.17	− 28.49**	0.196	0.061**	− 7.97	− 32.39**
ln Sunlight	− 5.91	− 16.39**	− 8.92	− 26.92**	0.241	0.059**	− 2.02	− 34.62**
ln Wind speed	− 7.44*	− 7.06**	− 8.86*	− 30.45**	0.344*	0.016**	− 8.76*	− 23.65**
ln Cem	− 8.16	− 11.01**	− 9.47	− 21.09**	0.126	0.044**	− 8.17	− 30.09**
ln Vpd	− 6.01	− 15.09**	− 9.02	− 25.02**	0.291	0.041**	− 2.92	− 31.69**

All tests, except KPSS, use the null hypothesis of the unit root (non-stationarity), while KPSS tests the hypothesis against the alternative of stationarity.

*1% level of significance.

**5% level of significance.

Published studies on fire-climate relationship^23,32^ generally do not resolve the non-stationarity issue that often arises in time series data that measures climate variables during recent years. Particularly, for explaining the variability of grassland area burned, ignoring the non-stationarity of the series leads to bad specified models that invalidate some of the results and generate high prediction error. In addition, modeling the series as if they were stationary may produce spurious relations that, although perceived as statistically significant, are not real and do not have a correct interpretation^33,34^. These potential problems generated by non-stationarity provide possible explanations for the large errors we observed in predictions and estimated relationships between area burned and climate. Cointegration is therefore a solution to model the complex dynamics of multivariate ecological time series when some of them are non-stationary. However, even under cointegration, all model parameters, including the lags of independent variables and the error correction term must be correctly specified. Moreover, the error term should not be serially dependent, in order to prevent spurious relationships arising from temporal correlations^33,35^. Implementation of statistical tests for stationarity of time series, known as unit root tests, is then a fundamental tool for the ecologist to analyze longitudinal data.

The ADF is among the most used and powerful unit root tests under constant variance and if the lag *p* is correctly selected. Alternatively, PP unit root tests differ mainly in how they address serial correlation and heteroskedasticity in the errors. ADF tests use a parametric autoregression to approximate the autoregressive moving averages structure of the errors in the test regression, whereas the PP tests ignore any serial correlation in the test regression. Unlike ADF tests, PP tests are robust to general forms of heteroskedasticity and do not require a specified lag length for the test regression^23^. The ADF and PP tests are asymptotically equivalent but may differ substantially in finite samples due to differences in correcting serial correlation in the test regression. In contrast, DF-GLS is a sensitive test that may increase the power of the previous, especially near stationarity and for small samples. Another recommendation, especially for short time series, is the simultaneous use of the KPSS test.

The lag selection criteria of log-likelihood^36^ (LR), the final prediction error^37^ (FPE), Akaike information criterion^38^ (AIC), Schwarz Bayesian criterion^39^ (SBC), and Hannan-Quinn information criterion^40^ (HQ) were used to select the optimal lag on all variables prior to testing for cointegration (see Supplementary Information Table S1). All these criteria are based on the minus maximized value of the respective likelihood function including a penalization term that increases with the number of estimated parameters to favor a more parsimonious model. Balancing out these two terms creates a metric that can be used as a model selection criterion. Both SBC and HQ are harsher in the selection than AIC, tending to choose a more parsimonious model. Therefore, we preferentially select the lag(1) as the optimal lag for subsequent model estimation in order to keep the model more compact and interpretable. The final prediction error, Schwarz Bayesian criterion, and Hannan-Quinn information criterion were used to confirm lag(1) as the optimal lag for subsequent model estimation.

The ARDL bounds testing approach is advantageous over traditional cointegration methods because it can examine a mixed order of integrations. We used the F-statistic to test for the null hypothesis of no-level relationship^41^ (SI Table S2). The bounds test for cointegration that the F-statistic was above both lower [1(0)] and upper [1(1)] bounds critical values, thus, all the models presented reject the null hypothesis of no cointegration. To validate the cointegration test, we further examined the null hypothesis following^42^ in the light of response surface regressions that report critical *p*-value approximations^43^. The *p-*values strongly rejected the null hypothesis at a 1% significance level, confirming a level relationship in the proposed models.

**Figure 3:**
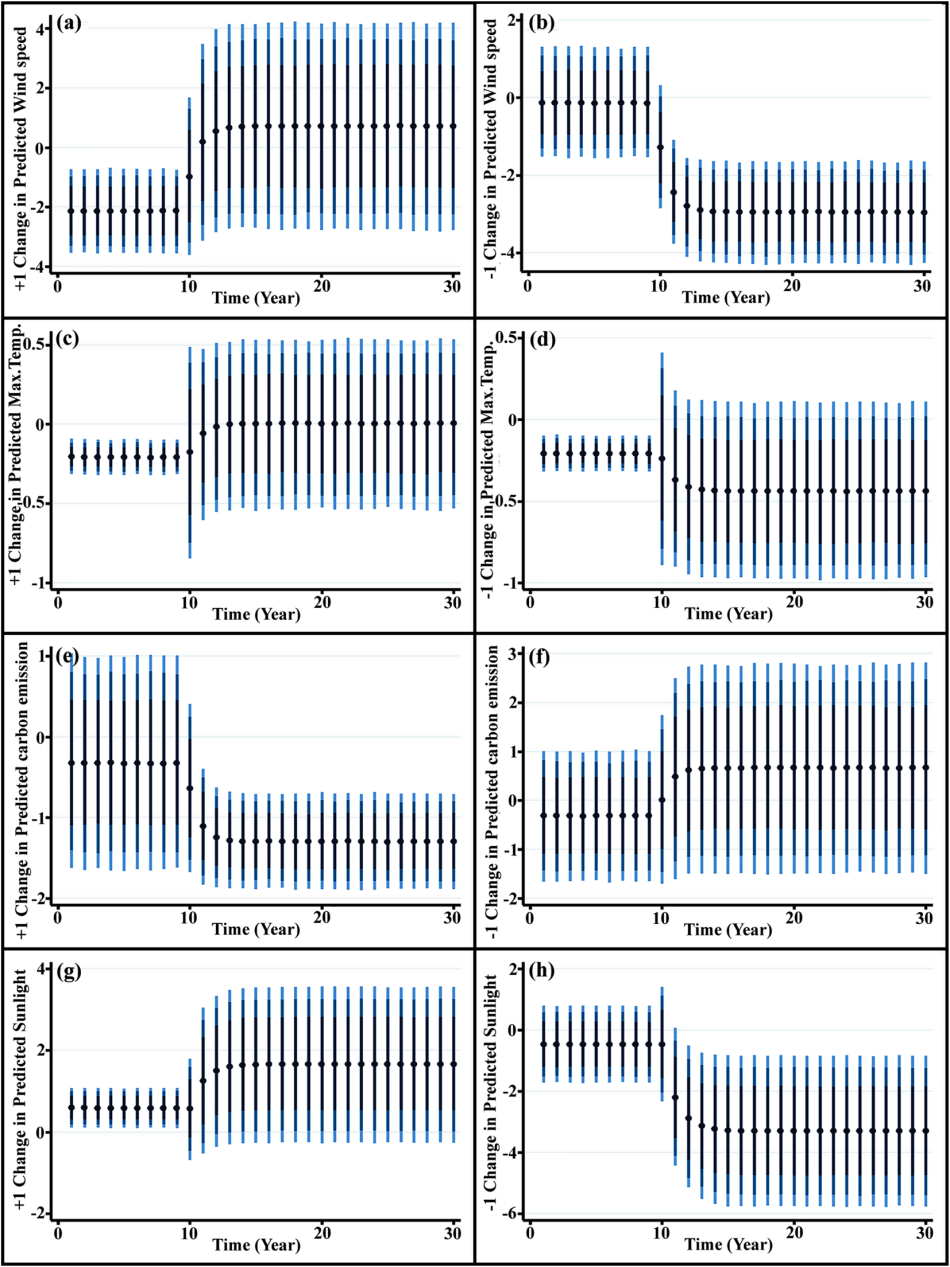
Plots of the dynamic simulated ARDL model showing the effect of: (**a**,**b**) positive and negative one standard deviation change in predicted wind speed on burned grasslands (**c**,**d**) positive and negative one standard deviation change in predicted maximum temperature on burned grasslands (**e**,**f**) positive and negative one standard deviation change in predicted carbon emission on burned grasslands (**g**,**h**) positive and negative one standard deviation change in predicted sunlight on burned grasslands. Dots represent average predicted value while dark blue to light blue lines denote 75, 90 and 95% confidence intervals. (For interpretation of the references to color in this figure legend, the reader is referred to the web version of this article.)

We implemented a graphical representation of the ARDL model simulation to provide an easily understandable representation of an instantaneous change (i.e., a shock) in one independent variable on the response in posterior observations (Fig. 3). This contrasts with the more elaborate interpretation of estimated coefficients, given their separation in long and short-run effects. For example, the dynamic plot shows that a positive shock (one standard deviation increase) at time t = 10 in wind speed produces a rapid positive short-run increase in burned grassland area.

At t = 15 this effect vanishes and the response value stabilizes thereafter (Fig. 3a). The confidence intervals (blue lines) indicate that the long-run change in burned grassland area is statistically significant using 75% confidence intervals, rather than significant with 90% confidence intervals. In contrast, the resulting plot of negative shock wind speed at time t = 10 decreased the mean response over several periods, resulting in a statistically significant equilibrium shift over four periods (Fig. 3b).

**Figure 4:**
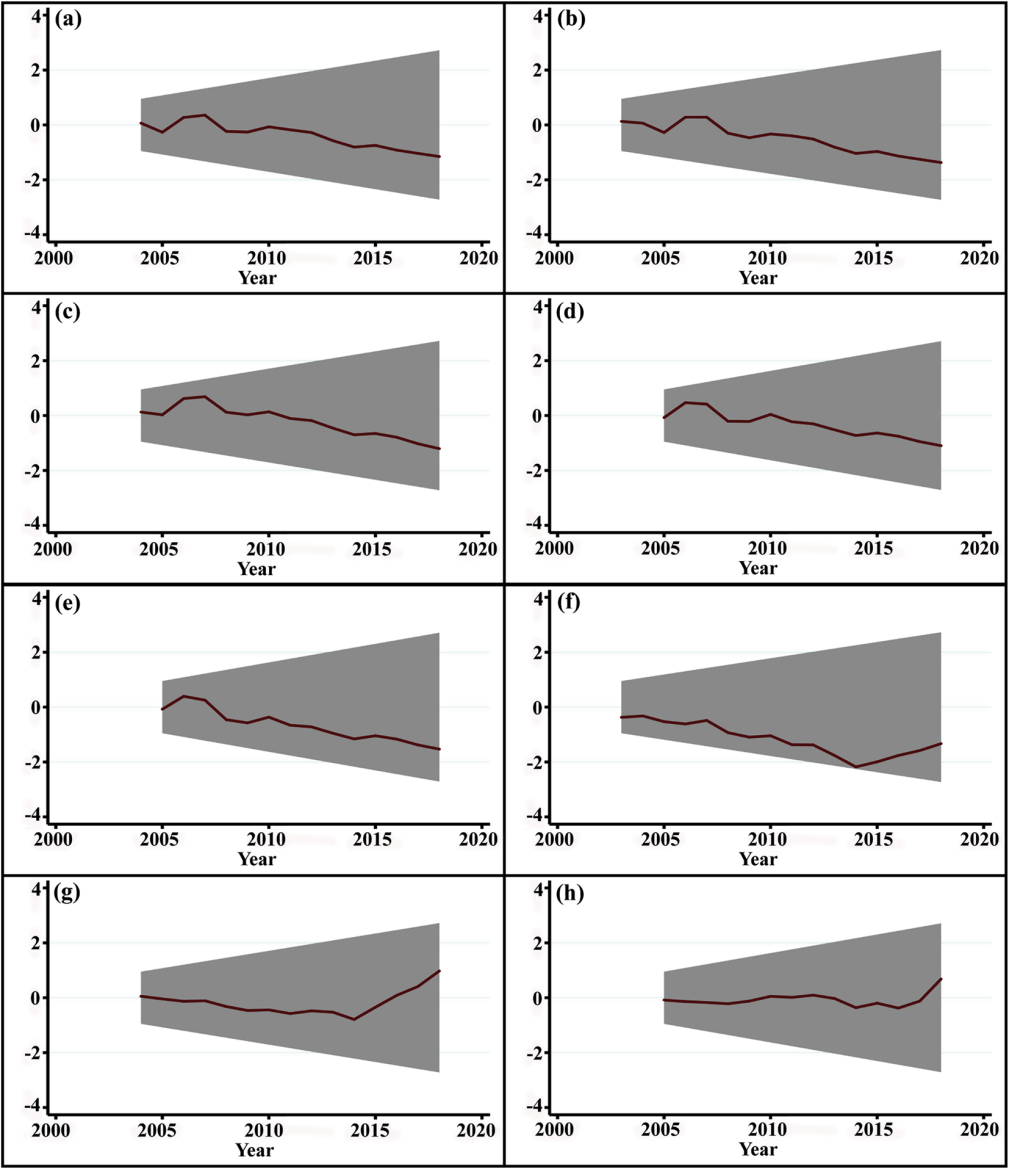
Recursive CUSUM plot of (**a**) maximum temperature, (**b**) minimum temperature, (**c**) sunlight, (**d**) wind speed, (**e**) vapor pressure deficit, (**f**) relative humidity, (**g**) precipitation, (**h**) carbon emission with 95% confidence bands around the null. For all variables, residuals fall between the limits, validating the ARDL bounds testing model and showing stability of the variances of coefficients over time.

To validate the model, we used diagnostic tests to examine the residual independence (Table 2). The coefficient of determination (R-squared) resulted in a goodness of fit of ~ 78.3%. The diagnostic tests showed that functional misspecification error, first-order serial correlation, heteroskedasticity, and non-normality assumptions are adequately controlled in the estimated model. To corroborate the independence of the residuals, we further employed the recursive cumulative sum (CUSUM) plots to examine the stability of the data series over the sampled period (Fig. 4).

**Table 2:** Short-run and long-run coefficients of dynamic ARDL.

Variable	Coefficient	Std. error	t-Statistic	Prob
ln *Wind*_*t*_	6.929*	1.591	4.352	0.01
∆ln *Wind*_*t*−1_	2.237*	0.106	21.06	0.01
ln *Tmax*_*t*_	2.361**	0.095	24.521	0.01
∆ln *Tmax*_*t*−1_	2.281*	0.519	4.393	0.01
ln *Rel.humidity*_*t*_	0.097	0.064	1.514	0.134
∆ln *Rel.humidity*_*t*−1_	0.068	0.067	1.034	0.305
ln *Precip*_*t*_	0.106	0.099	1.070	0.288
∆ln *Precip*_*t*−1_	0.091	0.103	0.883	0.380
ln *Sunlight*_*t*_	1.986*	0.412	4.814	0.01
∆ln *Sunlight*_*t*−1_	0.239*	0.112	2.128	0.01
ln *Tmin*_*t*_	0.043	0.083	0.519	0.605
∆ln *Tmin*_*t*−1_	0.073	0.126	0.578	0.564
ln *Vpd*_*t*_	0.180	0.155	1.161	0.249
∆ln *Vpd*_*t*−1_	0.017	0.064	0.271	0.786
ln *Cem*_*t*_	0.913**	0.155	5.872	0.01
∆ln *Cem*_*t*−1_	0.864*	0.189	4.562	0.01
ECT_*t*−1_	− 0.601*	0.124	− 4.81	0.000
*R*^2^	0.783			
Adjusted *R*^2^	0.714			
Simulations	5000			
Durbin–Watson-stat	2.082			
**Diagnostics**				
$\chi LM - ARCH^{2}$	0.38			
$\chi LM - B - G^{2}$	0.41			
Functional form	2.131 (0.121)			
Ramsey RESET test	3.421 (0.154)			
Normality	0.761 (0.681)			

ARDL (1,1,0,1,1,1,1) was selected based on the Schwarz Bayesian Criterion.

**1% level of significance.

*5% level of significance.

### Simulation and analysis of ARDL dynamics

The grassland ecosystems of Xilingol are characterized by highly dynamic and complex interactions among climate, human settlement patterns, ignition, and vegetation cover. Increased grazing constrains grassland burning leading to an “area burned deficit”. Otherwise, the system is highly responsive to high-speed seasonal winds with low relative humidity, quickly drying fuel, and increasing the potential for extensive grassland fires across the region^28^.

The long-run and short-run dynamics illustrated in Table 2 show that wind speed represents the dominant variable that extensively increases grassland area burned over both the long and short run. Arithmetically, a 1% increase in mean monthly wind speed increases the burned grassland area by 6.9% (different from zero in a 99% confidence interval (99% CI)) in the long run, and 2.2% (99% CI) in the short run, while holding other variables constant. Besides the direct impact of wind speed on the grassland area burned, it is likely to affect other environmental conditions which also influence fire behavior throughout the Xilingol region. That is, the dynamics of the whole system show complex causal relations that may not be completely explicit on single equation coefficients. For example, our data suggest that wind speed increased following the burning of grasslands (see Supplementary Information Fig. S1). This is consistent with the observation of more extensive burned grasslands over the study period also associated with a dramatic increase in wind speed, an indicator of potential positive feedback not considered in previous studies^23^. Importantly, high wind speeds were measured in May–August accompanied by the dry land surface conditions and atmospheric aridity, contributing to the influence of burning grasslands feeding back to further promote pyrogenic conditions. Above the threshold, each incremental increase in wind speed on a monthly scale leads to larger burned grasslands across the Xilingol region, overriding other ecological properties, and grassland functions.

### Threshold maximum temperature causes uncertainty in ecological change

The maximum temperature is also an important predictor of the increasing extent of burned grasslands. The estimated elasticity of maximum temperature in the long-run indicates that a 1% increase in maximum temperature results in a 2.4% increase in burned grassland extent (99% CI) while holding other variables constant (Table 2). The short-run estimated elasticity of maximum temperature is similar to the long-run response, as a 1% increase in maximum temperature leads to a 2.3% (99% CI) increase in burned grasslands. The results corroborate similar studies^13–15,22^.

The effect of maximum temperature could have more uncertainty because significant relationships between it and burned grasslands occur only when the regional summer maximum temperature exceeded a threshold level, producing a nonlinear relationship^22^. That is, slight changes in maximum temperature are unlikely to change the ecosystem except when a threshold is crossed, above which effects can be dramatic.

A more meaningful understanding of the effect of maximum temperature on burned grassland areas can be achieved by the use of the simulations of the ARDL model, given that all the relationships among variables are considered. At time t = 10, a small increase in maximum temperature in the short run is not statistically significant, but becomes statistically significant in the long run. Following a negative shock on the temperature at t = 10, the maximum temperature decreases, stabilizing three periods later. The equilibrium temperature shift over the long-run scenario time is statistically significant (95% CI). Discrepancies between burned grassland area and maximum temperature trends in Xilingol may be rooted in urbanization, vegetation type transitions, and/or natural variability to climate response. Additionally, the frequency of human ignitions, along with other anthropogenic disturbances, strongly supported the abrupt impact of maximum temperature overpowering the influence of other environmental variables (SI Fig. S2).

### Influence of sunlight on burned grasslands and ecological change

The amount of sunlight exacerbates changes in burned grassland area both directly via the drying effect of radiant heat on fuel and indirectly by influencing other climatic parameters such as temperature and humidity. A 1% increase in sunlight resulted in a 1.98% and 0.24% (99% CI) increase in burned grasslands in the long and short-runs, respectively (Table 2). A negative shock in ln(sunlight) at time (t = 10), insignificantly impacted burned grassland area over the next several periods (Fig. 3). This rapid response is explained by the inclusion of sunlight with a lagged-difference as a dependent variable. Conversely, a positive shock to ln(sunlight) at time (t = 10) increases area burned, which, in turn, shifts the equilibrium and predicts the ecological change just beyond t = 10.

### Impacts of burned grasslands to carbon emissions and ecological change

The consequence of carbon emissions from burned grasslands was found to be an important relationship in the long run. Carbon emissions, whether from these fires or other sources in Xilingol may influence the extent of burning in this region by contributing to the forcing of atmospheric temperature. More directly, grassland fires resets succession, potentially increasing ecosystem flammability (SI Fig. S3). The estimated elasticity of carbon emission, in the long run, is 0.91, hence, a 1% increase in carbon emissions is related to a 0.86% increase in burned grasslands (95% CI) in the long run and 0.91% (99% CI) in the short run (Table 2).

The graphical representation of Fig. 3 shows that a change in predicted carbon emissions on burned grasslands and ecological change. The positive shock of carbon emission on burned grasslands and ecological change was associated with a small increase in carbon emission in the short run that is statistically significant at time t = 10. Consistent with other modeled changes, carbon emissions also increase significantly over the predicted value in the long run.

### Ecological properties under threshold-governed ecosystems

The mechanism underpinning ecological properties can vary widely with significant uncertainty where threshold relationships among different biophysical processes occur^22^. The model suggests significant changes in the frequency of climatic variations prior to and in response to, the increased areal extent of burned grasslands (SI Figs. S1–S3). Our results highlight the importance of varying patterns in dominant burned grassland climatic factors (e.g., SI Figs. S1–S3) may also hint at broader implications, suggesting that sudden ecosystem state changes, fire-related or otherwise, may naturally exhibit significant variability across space and time^22^. The results strongly suggest that area burned increases mainly due to increases in wind speeds during the summer season when characteristically warm temperatures and low humidity facilitate fuel drying and fire spread (see SI Fig. S2) and not due to concurrent changes in anthropogenic activities such as fire suppression practices, land management, and human ignitions. This is not to say that anthropogenic activities are negligible in dictating the modern extent of annually burned grasslands. Rather, increased human ignition is associated with increased burned grassland extent, further confounding a modeling effort based solely on physical ecosystem drivers.

Our long-run analyses suggested that predicted burned grassland extent is insensitive to change in maximum temperature, vapor pressure deficit, precipitation, or relative humidity. These findings contrast with studies that predict increased fire impacts in north-eastern China in response to higher maximum temperature, decreased precipitation, greater vapor pressure deficit, and lower humidity^28,44–47^. We posit four potential explanations for this divergence: First, although inter-annual precipitation outside the fire season varies significantly in Xilingol grasslands, little precipitation arrives during the fire season itself. Therefore, inter-annual variation in fire season precipitation is insufficient to impact burned grassland extent. Second, grassland soil field capacity in this region is quite high. Therefore, fire season fuel moisture levels are accordingly unresponsive to precipitation variation. Third, the vast majority of fires in Xilingol originate from human activities^23,28^. These ignitions, and wind speeds and temperatures coincident with them, rather than fuel conditioning, may explain burned grassland extent. Finally, it is also possible that our statistical approach is insufficiently sensitive to detect a precipitation response under the conditions experienced across the 18-year time series used in this study.

### Model estimation

Our empirical strategy for estimating the proposed models follows a series of tests required before the application of the novel dynamic simulations of ARDL^27^. First, the dynamic ARDL simulations require that the series are integrated below order two, I(2), meaning that the series can be integrated of order one, I(1); integrated of order zero, I(0), or a mixture of both I(1) and I(0). To test the level of integration in the series, we utilized the Augmented Dickey–Fuller (ADF)^48^, Phillips–Perron (PP)^49^, Kwiatkowski, Phillips, Schmidt, Shin (KPSS)^50^, and modified Dickey-Fuller generalized least squares (DF-GLS)^51^ unit root techniques. While all the unit root techniques are tested based on the null hypothesis of unit root, KPSS is tested based on the null hypothesis of stationarity. Testing for unit root is an important component in the model estimation to prevent a spurious relationship between the series. A simplified version of testing for unit root can be expressed in AR (1) as:

1 $$y_{t} = a + \rho y_{t - 1} + \varepsilon_{t} ,\quad t = 1 \ldots T$$

where y_*t*_ is the series to be tested for unit root, ε_*t*_ is the error term. ε_*t*_ is independent and identically distributed, independent of the initial detected value y_0_, and has a zero-mean if the previous observation of $\mathrm{y}$ is: $E\left( {\varepsilon_{t} |y_{t - 1} \ldots y_{0} } \right) = 0$. The null hypothesis of the unit root can be tested by *H*_0_: *ρ* = 1 against the alternative *H*_1_: *ρ* < 1. If the null hypothesis is true, the model is non-stationary and would have an order of integration larger than 0. The ADF test includes lagged values of the response allowing for testing stationarity in higher-order autoregressive processes. The PP test makes a similar correction as ADF but uses a non-parametric modification of the *t* statistic that makes it more robust for unspecified autocorrelation and non-constant variance. The KPSS test may be used as a complementary test for stationarity due to its null hypothesis, which can be used with data on which other unit root tests are not conclusive. The DF-GLS test presents an improvement over ADF by using a generalized least squares rationale. On several empirical studies^52,53^ it has been shown to have better power performance for small-sample sizes and especially when the coefficient *ρ* is close to 1, that is, the series is near to non-stationarity.

After confirming the order of integration, the dynamic ARDL simulations require the series in the models to be cointegrated. The traditional concept of cointegration exists when a set of series are I(1) but there are some linear combinations of them that are I(0). This will, in effect, control for spurious regression that affects statistical inferences. Validation of the existence of cointegration confirms a possible long-run relationship between the series. Contrary to the traditional forms of cointegration that require all series to exhibit both I(0) and I(1), Pesaran proposed a vibrant cointegration method that deals with a sophisticated order of integration^41^. This means that the autoregressive distributed lag (ARDL) bounds testing cointegration do not require strict preconditions, except that the order of integration should not be I(2). Before cointegration was tested, we utilized lag selection criteria to determine the optimal lag. We used the ARDL bounds testing approach to examine the level relationship between the variables. The null hypothesis of no cointegration is tested using Fisher’s test^54^ statistic matched against the lower and upper bound critical values. The null hypothesis of no level relationship is rejection if the F-statistic is extreme compared to the upper bound critical values. To validate the bounds testing procedure for cointegration, we utilized the novel critical value bounds test and approximate p-values using response surface regression^43^.

Third, after validating the existence of a level relationship, the dynamic ARDL simulations require that the series are fitted with the error correction based-ARDL, expressed as:

2 $$\Delta y_{t} = \alpha_{0} + \delta_{0} y_{t - 1} + \delta_{1} x_{1,t - 1} + \cdots + \delta_{k} x_{k,t - 1} + \sum\nolimits_{i = 1}^{p} {\alpha_{i} \Delta y_{t - 1} } + \sum\nolimits_{j = 0}^{{q_{1} }} {\beta_{1,j} \Delta x_{1,t - j} } + \cdots + \sum\nolimits_{j = 0}^{{q_{k} }} {\beta_{k,j} \Delta x_{k,t - j} } + \varepsilon_{t}$$

where y_*t*_ denotes a change in the target variable, ${a}_{0}$ is the constant, y_t−1_ represents the lagged target variable at levels, $x$ represents the independent series in time (.)_*t*−1_ at levels, $\mathrm{a}??$ is the first difference, $p$ and $q$ are the optimal lags, and ε_t_ is the error term. Model 2 corresponds to a particular formulation called Error Correction Model (ECM), where long and short-run effects are combined. Parameters δ_*j*_ for j = 0, 1,…, k; are related to the long-run relations that were found by cointegration. Expressing and for j = 1,…, k, then all variables related to δ coefficients maybe rearranges as: $\left( {\alpha_{0} y_{t - 1} + \alpha_{1} x_{1,t - 1} + \cdots + \alpha_{k} x_{k,t - 1} } \right) = \alpha_{0} EC_{t - 1}$ where ${EC}_{t-1}$ is known as the error correction term, that allows to estimating parameters in Model 2 given cointegration.

The estimated models were validated using the cumulative sum and cumulative sum of square^55^, to examine the stability of the estimated models. After meeting all the pre-conditions, the novel dynamic simulations of ARDL can then applied using Eq. (2) but modified to include counterfactual change using a single shocked regressor, amount of shock to be applied (±), length of the scenario for the simulation, shock scenario time, number of simulations, burn-in rate, and the resulting dynamic plot.

## Conclusion

This study demonstrates the importance of addressing non-stationarity of time series data, using the unit root test to elucidates short- and long-term ecosystem responses. Models for fire-prone grassland, and other landscapes existing near-threshold levels for key ecosystem drivers are susceptible to compounding data inaccuracies. The dynamic simulated ARDL approach identified wind speed as the most important climatic variable in determining the model outputs in the complex fire-climate-human relationship, followed by maximum temperature, with sunlight and carbon emission having only minor to modest influence. Potential positive and negative feedbacks were identified and these are needed to inform best management practices. Adaptation of this approach would help generate the more precise information needed by ecologists and stakeholders to plan and respond appropriately to management challenges in fire-prone landscapes under future environmental scenarios.

## Supplementary information

Supplementary Information.

## Data Availability

The data of climatic variables from 2001 to 2018 were taken from the China Meteorological Data Sharing Service Centre (https://data.cma.cn/en; accessed 28 June 2020). Xilingol burned grasslands records were collated from the Monitoring Centre of the Ministry of Agriculture (https://www.moa.gov.cn/; accessed 28 June 2020). The field data of Xilingol burned grasslands are available from the corresponding author upon request.
