# Supplementary material for: Seasonal weather and climate prediction over area burned in grasslands of northeast China
Source: Sci Rep. 2020 Nov 17;10:19961. doi: 10.1038/s41598-020-76191-2 (PMC7672083; doi:10.1038/s41598-020-76191-2)
Supplement: Supplementary file 1 — Supplementary Information. [file 41598_2020_76191_MOESM1_ESM.doc]

**Seasonal weather and climate prediction over area burned in grasslands of northeast China**

Ali Hassan Shabbir^1,2,3,+^, Jiquan Zhang^1,2,3,*^, John W. Groninger^4,+^, Eddie J. B. van Etten^5,+^, Samuel Asumadu Sarkodie^6^, James A. Lutz^7,+^, Carlos F. Valencia^8^

**Supplementary Information**

**Tables:**

**S1 Selection criteria of lag order of variables for the Autoregressive Distributed Lag (ARDL) approach.**

| Lag | LogL | LR | FPE | AIC | SBC | HQ |
| --- | --- | --- | --- | --- | --- | --- |
| 0 | 131.06 | NA | 8.11e+11 | -8.71 | -9.11 | -6.91 |
| 1 | 119.02 | 276.21 | 1.21e+11* | -14.34 | -15.04* | -22.21* |
| 2 | 129.10 | 63.75 | 6.02e+11 | -13.01 | -16.91 | -23.67 |
| 3 | 114.14 | 91.02* | 9.01e+11 | -16.42* | -18.61 | -24.64 |

Notes: * indicates lag order selected by the criterion, LogL: log-likelihood, LR: sequentially modified log-ratio test statistic (each test at 5% level), FPE: Final prediction error, AIC: Akaike information criterion, SBC: Schwarz Bayesian Criterion and HQ: Hannan-Quinn information criterion.

**S2 ARDL bounds cointegration test results.**

| Models | statistic |  | 1(0) | 1(1) | p-value  1(0) | 1(1) |
| --- | --- | --- | --- | --- | --- | --- |
| 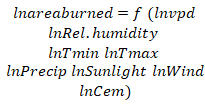 | F | 11.21 | 2.64 | 3.66 | 0.001 | 0.001 |
| 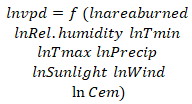 | F | 9.11 | 2.92 | 3.81 | 0.001 | 0.001 |
| 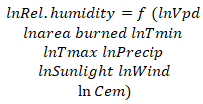 | F | 8.95 | 2.48 | 3.47 | 0.002 | 0.001 |
| 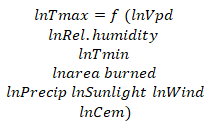 | F | 6.51 | 2.66 | 3.36 | 0.001 | 0.001 |
| 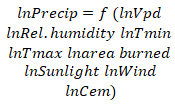 | F | 10.43 | 2.54 | 3.85 | 0.008 | 0.007 |
| 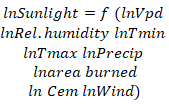 | F | 5.91 | 2.81 | 4.38 | 0.001 | 0.001 |
| 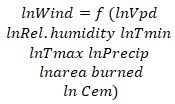 | F | 8.24 | 2.71 | 3.51 | 0.001 | 0.001 |

**Figures:**

**
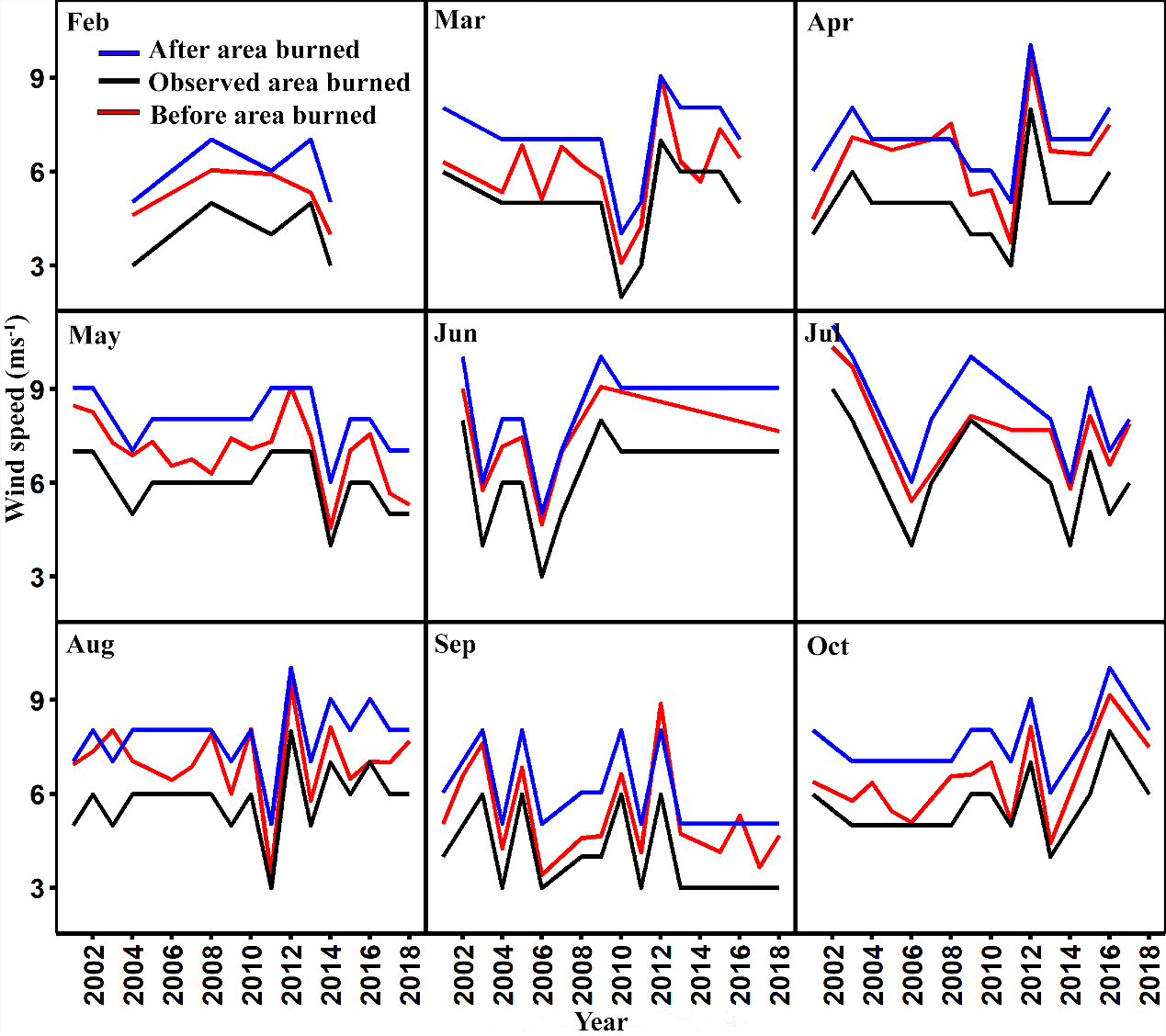
**

**S1** The wind speed colors indicate burning grassland area of 2000-2018. The blue color representing the condition of wind speed after burning grassland area, black color representing observed burning grassland area and red color representing observed burning grassland area. There were no fires in January, November, or December between 2001 and 2018.

**
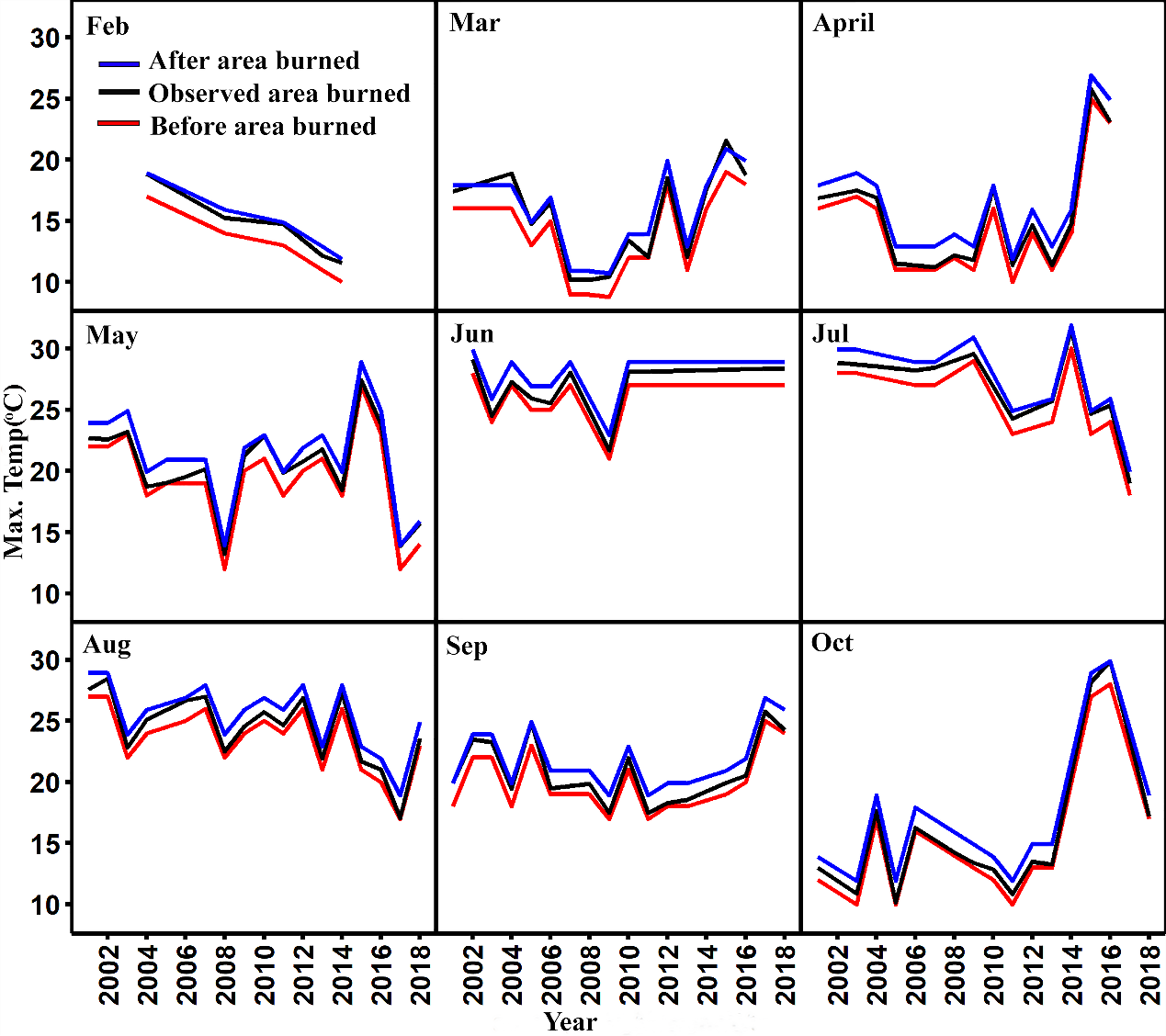
**

**S2** The maximum temperature colors indicate burning grassland area of 2000-2018. The blue color representing the condition of maximum temperature after burning grassland area, black color representing observed burning grassland area and red color representing observed burning grassland area. There were no fires in January, November, or December between 2001 and 2018.

**
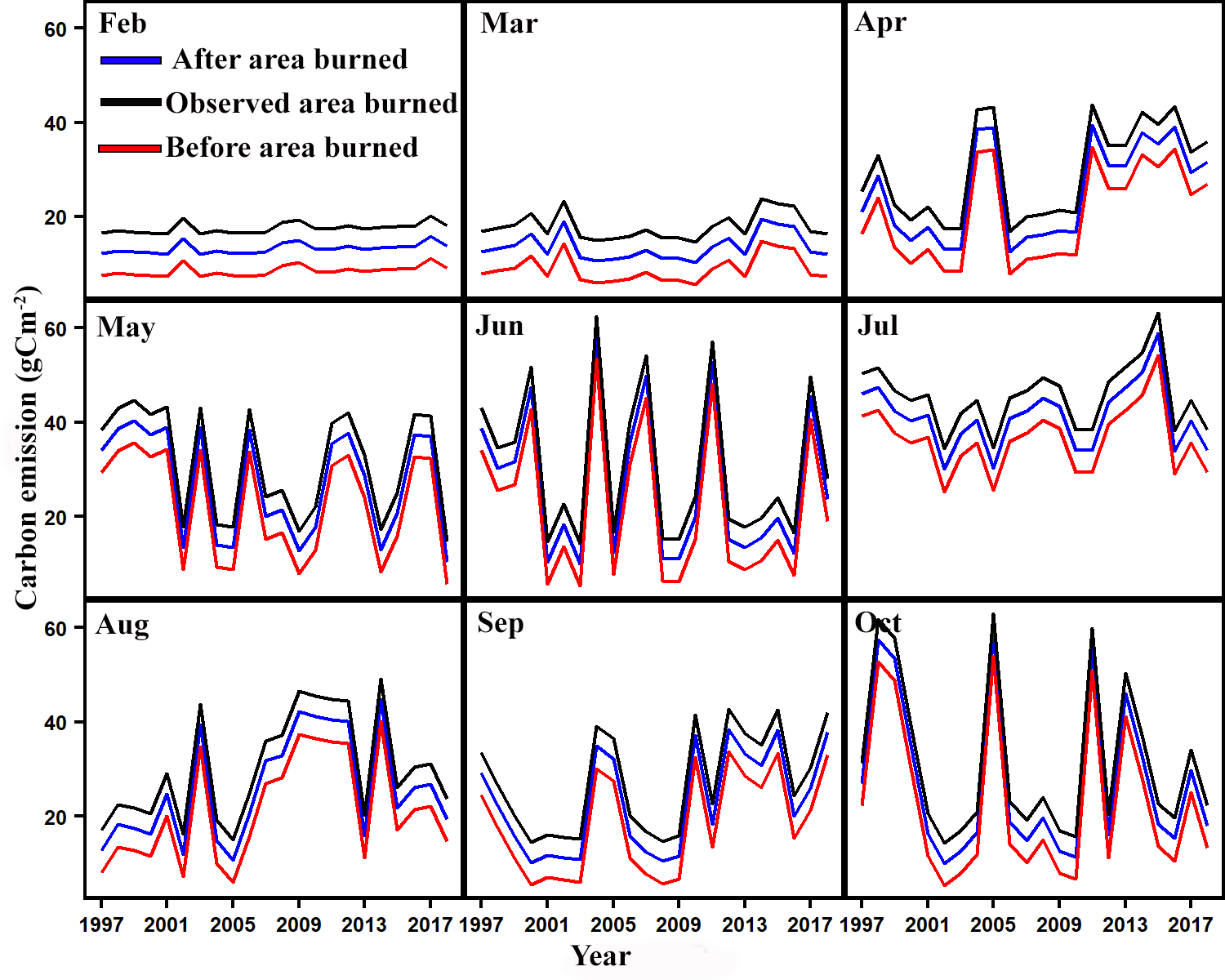
**

**S3** The carbon emission colors indicate burning grassland area of 2000-2018. The blue color representing the condition of carbon emission after burning grassland area, black color representing observed burning grassland area and red color representing observed burning grassland area. There were no fires in January, November, or December between 2001 and 2018.

**
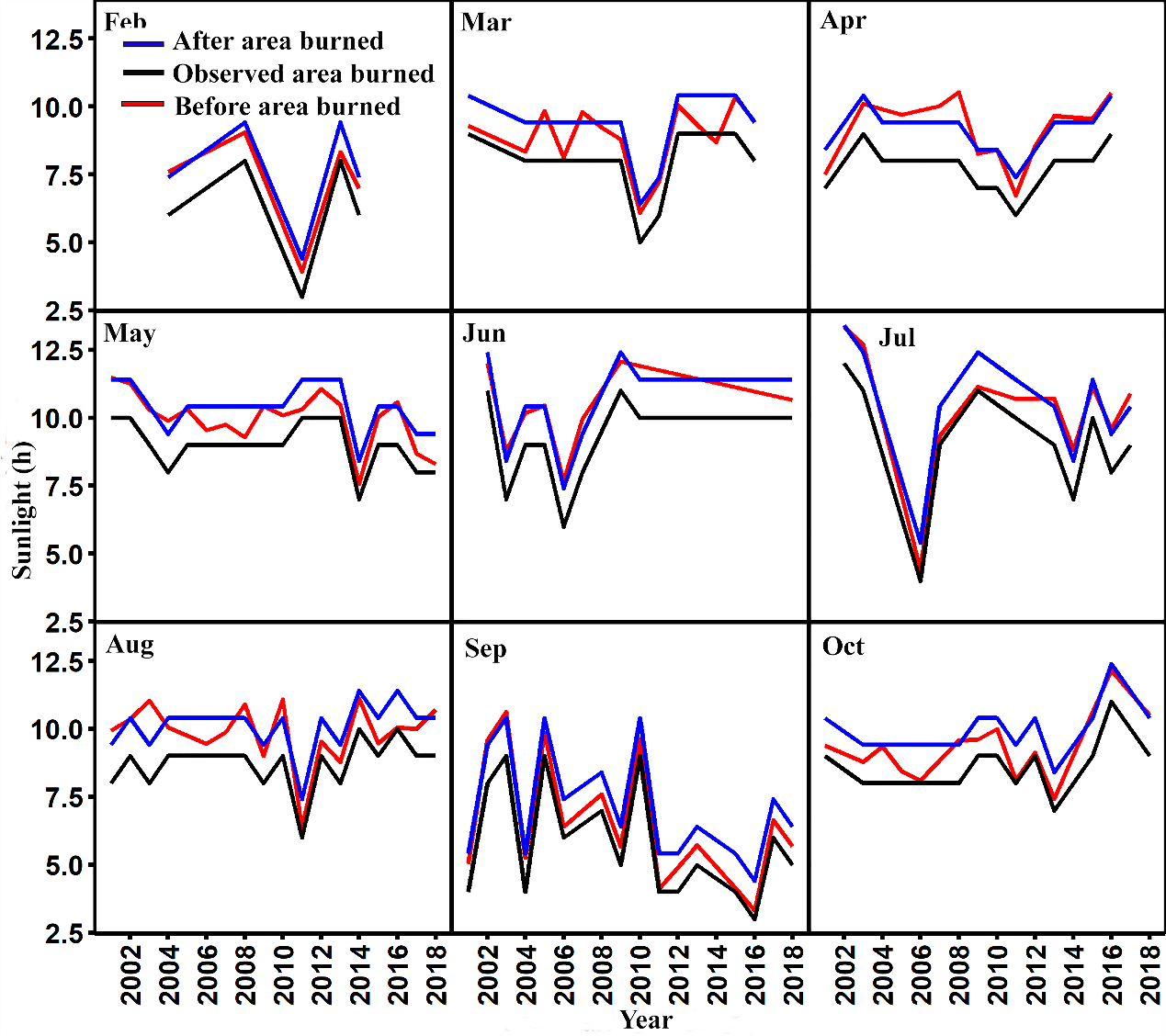
**

**S4** The sunlight colors indicate burning grassland area of 2000-2018. The blue color representing the condition of sunlight after burning grassland area, black color representing observed burning grassland area and red color representing observed burning grassland area. There were no fires in January, November, or December between 2001 and 2018.
